# Recent Advances in Nanomaterials for Asthma Treatment

**DOI:** 10.3390/ijms232214427

**Published:** 2022-11-20

**Authors:** Xu Zuo, Xiaoping Guo, Yinuo Gu, Haoyu Zheng, Zhengjie Zhou, Xinlei Wang, Shengyu Jiang, Guoqiang Wang, Caina Xu, Fang Wang

**Affiliations:** 1 Department of Pathogeny Biology, College of Basic Medical Sciences, Jilin University, Changchun 130021, China; 2 Department of Biochemistry, College of Basic Medical Sciences, Jilin University, Changchun 130021, China; 3 Key Laboratory of Pathobiology, Ministry of Education, Jilin University, Changchun 130021, China

**Keywords:** biomedical polymers, asthma, nanoparticles, drug delivery, nanomaterials

## Abstract

Asthma is a chronic airway inflammatory disease with complex mechanisms, and these patients often encounter difficulties in their treatment course due to the heterogeneity of the disease. Currently, clinical treatments for asthma are mainly based on glucocorticoid-based combination drug therapy; however, glucocorticoid resistance and multiple side effects, as well as the occurrence of poor drug delivery, require the development of more promising treatments. Nanotechnology is an emerging technology that has been extensively researched in the medical field. Several studies have shown that drug delivery systems could significantly improve the targeting, reduce toxicity and improve the bioavailability of drugs. The use of multiple nanoparticle delivery strategies could improve the therapeutic efficacy of drugs compared to traditional delivery methods. Herein, the authors presented the mechanisms of asthma development and current therapeutic methods. Furthermore, the design and synthesis of different types of nanomaterials and micromaterials for asthma therapy are reviewed, including polymetric nanomaterials, solid lipid nanomaterials, cell membranes-based nanomaterials, and metal nanomaterials. Finally, the challenges and future perspectives of these nanomaterials are discussed to provide guidance for further research directions and hopefully promote the clinical application of nanotherapeutics in asthma treatment.

## 1. Introduction

Asthma is a chronic airway inflammatory disease, which is characterized by airway hyperresponsiveness (AHR), eosinophil infiltration, mucus hypersecretion, airway remodeling, reversible airflow obstruction, and goblet cell proliferation. The clinical manifestations are recurrent cough, wheezing, and dyspnea [1]. Asthma can be triggered or aggravated by a number of factors, including immune stimuli such as allergen-specific immunoglobulin E (IgE), as well as non-immune stimuli caused by exercise or certain medications [2]. In addition, genetics and obesity are also major sources of asthma. By 2025, the global asthma population may reach 400 million [3]. Asthma is classified in several ways. In recent years, the classification that has attracted attention is mainly based on the types of inflammation and immune cells involved, which can be divided into T helper 2 (Th2)-high and Th2-low subtypes [4,5]. The Th2-high subtype is characterized by marked eosinophilic infiltration of the airway, while the Th2-low subtype is characterized by neutrophil infiltration [6,7]. Cytokines such as interleukin (IL)-4, IL-5, and IL-13 released by Th2 cells are dominant in the progression of Th2-high subtype of asthma, and their secretion is closely related to bronchial inflammation, smooth muscle spasm, and excessive secretion airway mucus. It also leads to irreversible airway remodeling, excessive fibrosis, and scarring of collagen deposition, exacerbating the disease process [8]. Th2-low subtype of asthma, also known as severe asthma, is primarily mediated by the T helper 17 (Th17) signaling pathway [4]. Th17 cytokine levels have been shown to be significantly elevated in the tissues of patients with severe asthma and have been identified as a key factor in severe asthma [9]. Regardless of the type of asthma, it is important to rapidly reduce inflammation in the airways and prevent the false activation of allergen hypersensitivity mechanisms.

The treatment of asthma is mainly based on drug therapy. Inhaled glucocorticoids are the mainstay of treatment. After the specific binding of glucocorticoids to glucocorticoid receptors, it can reduce the expression of pro-inflammatory factors, inhibit the excessive activation of signaling pathways such as nuclear factor kappa-B (NF-κB) and mitogen-activated protein kinase (MAPK), and inhibit the increase of inflammatory cells to relieve asthma [10,11,12]. However, long-term use of glucocorticoids can cause drug resistance and many side effects, such as osteoporosis, growth suppression in children, and diabetes [13,14]. In recent years, considering patients’ medication compliance, glucocorticoids have been used in combination with other drugs in small doses to better prevent side effects [10,15]. In addition, theophylline can reduce airway inflammation as it restores steroid sensitivity and increases histone deacetylase 2 (HDAC2) [16,17]. However, theophylline has many side effects, such as causing nausea, headache, and epilepsy in the body [18]. Moreover, long-acting beta agonists/muscarinic antagonists are also commonly used in the treatment of asthma, and they can suppress the inflammatory response by inhibiting thymic stromal lymphopoietin messenger RNA (mRNA) in the bronchi [19,20]. With the rapid development of science and technology, more and more therapies have been developed for the treatment of asthma. Long-acting β2-receptor agonists are the drugs of choice for the treatment of adolescent asthma [21]. In recent studies, leukotrienes, as key lipid mediators of allergic diseases, have also been considered as new targets for asthma treatment [22]. Leukotrienes trigger inflammation during allergic reactions in the airways, and leukotriene receptor antagonists may have therapeutic effects by inhibiting increased mucus secretion, bronchoconstriction, and airway inflammation [22,23]. Monoclonal antibody-derived therapies are gaining recognition. Several monoclonal antibodies have been or are undergoing in clinical trials, including anti-IgE antibodies (omalizumab), IL-33R antibodies (CNTO 7160), anti-IL-4Rα antibodies (dupilumab), and anti-IL-5 antibodies (mepolizumab and reslizumab) [2]. Antihistamines and allergy medicines also play an important role in asthma treatment. Specific blockers of histamine receptors improve lung inflammation by interrupting histamine signaling [24,25]. Antiallergic drugs such as loratadine or cetirizine are well tolerated as adjunctive therapy for asthma [26]. Notably, some antihistamines can cause side effects such as drowsiness and new cardiac arrhythmias and are not allowed in some countries [27]. Although the introduction of new therapies recent years has been improved the therapeutic effect of asthma, the treatment effect varies greatly due to individual differences in patients [8]. Therefore, there is an urgent need to develop effective treatments for asthma. 

**Figure 1:**
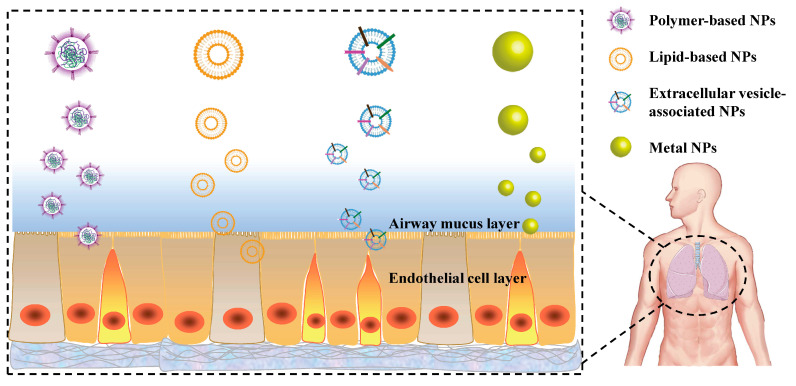
Variety of nanoparticles (NPs) used in the treatment of asthma.

In recent years, nanotechnology has been widely developed in modern medicine and pharmacy applications. Nanomaterials not only have obvious advantages in improving pharmacokinetics, prolonging blood circulation time and reducing drug toxicities, but also in enabling targeted drug delivery, slowing the release of drugs, and enhancing drug solubility [2]. Nanomaterials have become a research hotspot for researchers in many fields due to their unique physicochemical properties, such as controllable size, good biocompatibility and low cytotoxicity [28]. Many nanomaterials and micromaterials, such as polymeric nanoparticles, solid lipid nanoparticles, extracellular vesicles nanomaterials and metal nanoparticles have been explored in asthma treatment (Figure 1). Among them, polymeric nanoparticles are the most widely used due to their high modifiability and many other advantages, making them the most popular and effective drug delivery materials. Nanoparticles not only improve the bioavailability of hormonal drugs and reduce the number of hormonal drug administrations, but also provide an outstanding contribution to the treatment of asthma by acting as a delivery agent for protein and nucleic acid drugs, avoiding their rapid degradation in the body. In this review, we present the use of nanomaterials and micromaterials in asthma, and hope that will trigger some new ideas and stimulate more efforts to promote the widespread use of nanotechnology in asthma treatment.

## 2. The Application of Different Kinds of Carriers in Asthma Treatment

### 2.1. Application of Polymetric Carriers in Asthma

**Figure 2:**
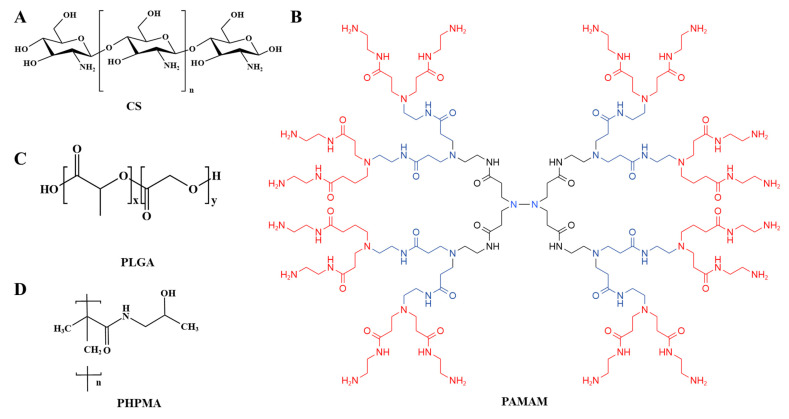
Structure of polymers for the treatment of asthma: (**A**) CS, (**B**) PAMAM, (**C**) PLGA, (**D**) PHPMA.

Polymers, consisting of monomeric units linked by covalent bonds, are the most diverse and broadly used class of biomaterials [29]. They have attracted great interest as drug delivery vehicles due to their good pharmacokinetics, long circulation times and desirable biocompatibility and biodegradability properties [30]. By modifying polymer-based drug carriers with targeting ligands, polymers could be used in drug delivery systems in the form of polymeric drugs for precise delivery of drugs. Herein, various types of polymers were used as drug delivery systems for the treatment of asthma, mainly involving chitosan (CS), Poly (D, L-lactide-co-glycolide) (PLGA), Poly (2-hydroxypropyl) methacrylamide (PAMAM) and poly (N-(2-hydroxypropyl) methacrylamide) (PHPMA) (Figure 2). The following was the summary of the characteristics of the above polymers and their use in asthma (Table 1).

#### 2.1.1. The Application of CS in Asthma

CS is a linear polysaccharide with a large number of amino groups generated from the deacetylation of chitin, deriving from the exoskeleton of crustaceans, which is a relatively abundant biopolymer in nature [56]. Due to its high permeability, biodegradability and good compatibility, there is a growing interest in various industries, especially in the modern pharmaceutical area [57,58,59]. CS has been explored in pharmaceutical, biotechnology, tissue engineering, etc., and researchers found that it has a huge number of pharmacological actions, such as antimicrobial, antioxidant, anti-inflammatory, anticancer, wound-healing, bone tissue-engineering, regenerative medicine, and mucosal adjuvant [60,61,62,63]. As the nanomaterial, several studies have demonstrated the safety of CS not only in the human airway cell culture models (CALU-3 cells and A549 cells), but also in many animal experiments by different routes of administration [64,65]. Due to its compatibility with airway epithelium cells, good mucoadhesion, and antibacterial properties, CS is widely used in novel nanotherapeutic drug development for asthma [57,66,67].

**Figure 3:**
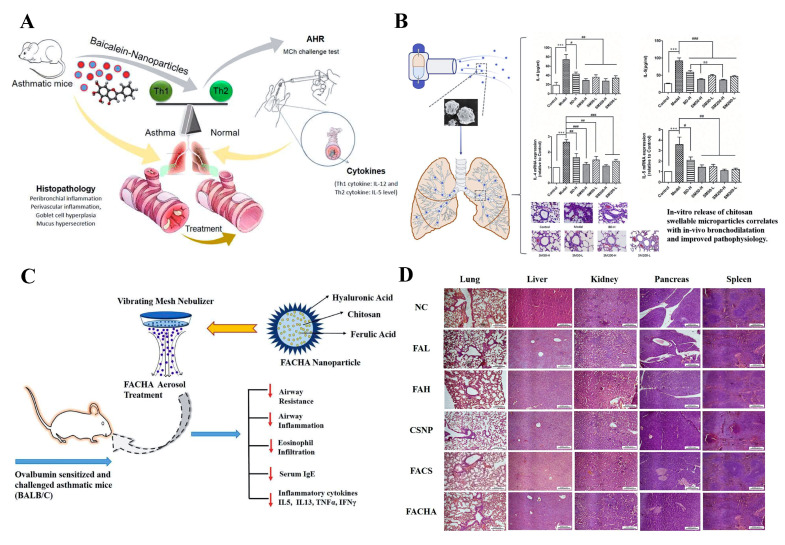
(**A**) The application of chitosan loaded baicalein nanoparticles (L-B-NPs) for asthma treatment. Reproduced with permission from [31], © 2022 The Author(s). Published by Elsevier B.V. on behalf of King Saud University. (**B**) The chitosan-based swellable microparticles for loading budesonide reduced the levels of IL-4 and IL-5 in-vivo and improved pathophysiology. Reproduced with permission from Statistics: aa *p* <0.01; *** *p* <0.001 vs. control group; ### *p* < 0.001, ## *p* < 0.01, # *p* < 0.05 vs. model group [34], © 2022 Elsevier B.V. (**C**) HA decorated, FA loaded CS nanoparticles (FACHA NPs) were administered by nebulization and exerted a therapeutic effect on OVA-sensitized and challenged asthmatic mice. (**D**) FACHA NPs did not cause significant damage to the lungs, liver, kidneys, pancreas or spleen in vivo. Reproduced with permission from [36], © 2022 Elsevier B.V.

One of the most attractive features of CS for use in asthma treatment is its mucus adhesion and penetration enhancing properties [68]. The basic amino groups of CS are protonated positively charged [69]. Negatively charged mucins are abundant in the mucus layer of the trachea [57,70]. Each mucin monomer is 0.2–0.6 µm in length and the monomers are linked together end-to-end by disulphide bonds [71]. Therefore, the positive charge of CS provides mucoadhesive properties, and promotes adhesion to mucus through positive and negative electrosorption [70,72]. The inflammatory immune response caused by asthma leads to obstruction of the airway and mucous production causes limitation of air flow [73,74]. To enhance the mucus permeability of baicalein, chitosan-loaded baicalein nanoparticles (L-B-NPs) were prepared. L-B-NP could control the pathophysiology of asthma by regulating the Th1/Th2 balance which could control the asthma pathophysiology by modulating the Th1/Th2 homeostasis (Figure 3A) [31]. In addition, the chitosan-based swellable microparticles for loading budesonide were prepared by a spray-drying method, which could effectively prolong the contact between the drug and the mucosa, reduce the number of patients taking the medicine, prolong the interval between administrations, and improve the patient’s compliance. After seven days of treatment, the number of eosinophils in the lung tissue was further reduced, and the levels of IL-4 and IL-5 in the bronchoalveolar lavage fluid and lung tissue were significantly reduced (Figure 3B) [34,37]. In addition, CS nanocarriers have achieved remarkable therapeutic effects in preventing the rapid clearance of nasal mucociliary and overcoming the limitation of low permeability across the mucosal barrier [35]. For example, soluble IL-17RC protein, a co-receptor subunit of IL-17 and IL-17F, inhibits downstream signaling to achieve anti-inflammatory effects; however, direct inhalation of it has no significant effect [35,75]. Inhalation of CS- recombinant protein IL-17RC (CS-RC) nanoparticles reduced airway inflammation in Th2-low endotype asthma, which is referred to as “severe asthma”, primarily based on neutrophil infiltration and the IL-17 pathway [76,77,78]. When CS opens the tight junctions contact between airway epithelial cells, protein-loaded CS has the potential to cross the ciliary layer of nasal mucosa [35]. As a natural polysaccharide with biocompatibility, in addition to the functions mentioned above, CS-based nanoparticles can protect nucleic acid components from being destroyed by nucleases in the body. It plays an outstanding role in allergen-specific immunotherapy (desensitization), which promotes the transmembrane absorption of proteins and peptides, and plays a safe and reliable role in the transmission of allergens [32].

CS can change the morphology of nanoparticles and increase the stability of nano-system, making a remarkable contribution to the biomedical development of different kinds of nanoparticles. Ferulic acid (FA) has potent free radical scavenging ability, but its poor permeability and extremely short half-life (30 min) largely limit its therapeutic applicability [36]. The limitations of FA were optimized by loading CS and decorating hyaluronic acid (HA), which ensured the protection and transport of FA across the lung barrier without causing significant damage to the lung, liver, kidney, pancreas and spleen in vivo (Figure 3C, D) [36]. Additionally, other researchers developed CS-based nanocarriers encapsulated with heparin to improve their stability, effectively preventing them from being eroded by various enzymes in the respiratory tract. The encapsulated heparin nanoparticles interacted with rat mast cells to achieve asthma therapeutic effects by reducing inflammation and airway remodeling [33]. Nanoparticles prepared based on CS not only optimize the stability of nanoparticles, but also have the following advantages, such as increased cellular uptake, tissue mucoadhesion and penetration, controlled drug release and improved antimicrobial effects [56]. In addition, CS as a coating material does not alter the rate of drug release but affects the mode of drug release, and it has been shown that polymeric nanoparticles coated with CS can be used to release drugs in acidic pH values [79,80]. Therefore, it can be based on the fact that CS can be used to control drug release by interacting with biological fluids, salts and different delivery media.

**Figure 4:**
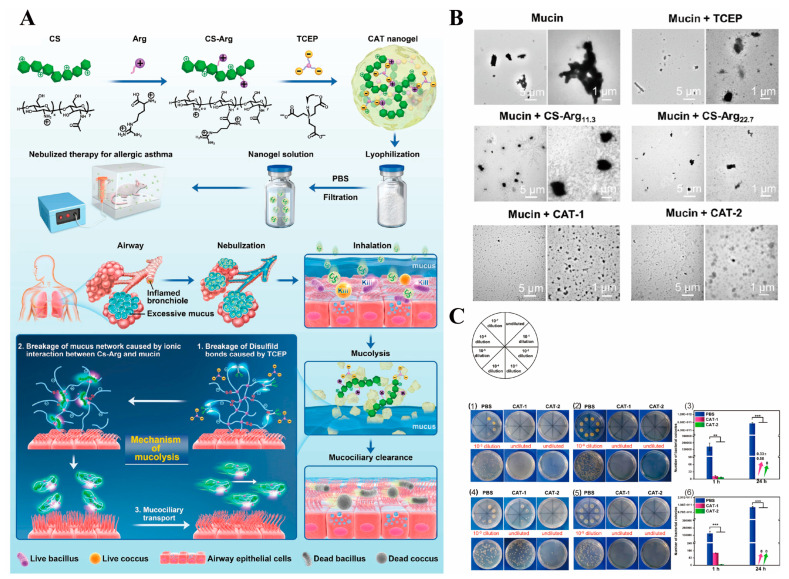
(**A**) The treatment of CS-Arg/TCEP nanogels for asthma. (**B**) TEM images of mucin and mixtures of mucin with different samples. (**C**) Antibacterial experiments of CS-Arg/TCEP nanogels. Reproduced with permission from (1) 1 h and (2) 24 h pictures of the inhibition of different samples against *S. aureus* aureus. (3) Quantitative of (1-2). (4) 1 h and (5) 24 h pictures of the inhibition of different samples against *E. coli* aureus. (6) Quantitative of (4-5). Statistics: ** *p* < 0.01, and *** *p* < 0.001. [38], Copyright © 2022 American Chemical Society.

In recent studies, the antimicrobial properties of CS played an important role in the synthesis of nanogels for the treatment of asthma. Mucus obstruction can lead to bacterial infection and chronic inflammation in the airways [81]. High levels of bacteria such as *Haemophilus*, *Neisseria*, *Streptococcus* and *Staphylococcus* have been reported to be present in the respiratory tract of asthmatic patients [82]. Zhao et al. developed the nanogel consisting of tris(2-carboxyethyl) phosphine (TCEP) and Arg-grafted CS (CS-Arg) for the treatment of asthma (Figure 4A) [38]. On the one hand, TCEP could cause disulfide bond cleavage. On the other hand, the ionic interaction of CS-Arg with mucin resulted in the disruption of the mucus network. After interaction of CS-Arg, TCEP and nanogels with porcine gastric mucin, a significant reduction in the size of the aggregates of porcine gastric mucin was observed, indicating that the nanogel could disrupt porcine gastric mucin (Figure 4B). In addition, CS-Arg had the ability to target the bacterial anion and significantly inhibited the growth of *S. aureus* and *E. coli* (Figure 4C). Nanogels as hydrophilic polymers can avoid the use of organic solvents, and also have the advantage of combining a small molecule reducing agent with large molecule therapeutic agents. Therefore, nanogels with good biocompatibility can be used as carriers to deliver various drugs for future asthma treatment applications.

#### 2.1.2. The Application of PLGA in Asthma

PLGA is approved for medical use by the U.S. Food and Drug Administration (FDA) and the European Medicines Agency (EMA), and widely used in drug delivery systems to encapsulate hydrophilic and hydrophobic drugs [83,84]. Its excellent biocompatibility, biodegradability, and unique physical and chemical properties make it one of the most popular and effective polymers for drug delivery [83,85,86]. The main products of PLGA decomposition are lactic acid and glycolic acid, which are easily metabolized and cleared by the body and thus are popular in the development of novel nanoparticles for the treatment of asthma [87].

PLGA as carrier has many advantages in the treatment of asthma, such as improving the bioavailability of hydrophobic drugs and achieving sustained release [40,41,49]. Inhaled hormone drugs play an important role in the treatment of lung inflammation caused by asthma, but most hormone drugs are hydrophobic in nature [40]. Preparation of PLGA-based porous polymer microparticles was completed using an oil-in-water double-emulsion method with ammonium bicarbonate as the porogen agent, which could effectively deliver budesonide, achieving the continuous delivery and dose reduction of budesonide [40]. Another study showed that, the density and porosity of PLGA particles could be altered by using polyethylenimine (PEI) porogen. It can increase deep lung deposition and lung retention of montelukast encapsulated in PLGA particles [44]. In addition, the preparation of PLGA nanoparticles improved the bioavailability of hormonal drugs, which could be released slowly, reduce the number of doses, and reduce systemic adverse effects such as osteonecrosis, oral fungal infections and osteoporosis [44,88,89].

**Figure 5:**
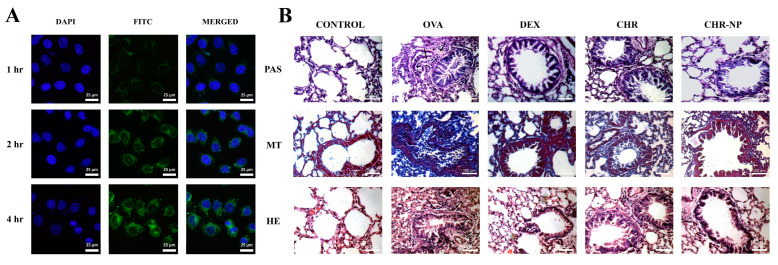
(**A**) Immunofluorescence images of A549 cells. (**B**) Exemplar photomicrograph of lung tissues from all the experimental groups stained by periodic acid-Schiff (PAS), Masson’s trichrome (MT), and hematoxylin-eosin (H&E) staining. Reproduced with permission from [49], © 2022 Elsevier Inc.

PLGA was widely used not only in the delivery of hormones, but also made progress in the delivery of herbal extracts. The herbal extracts extracted from natural medicines have the advantages of low toxicity and fewer side effects. However, the bioavailability and bioactivity of these extracts are limited by poor water solubility and rapid metabolism. Moreover, most herbal extracts do not have certain cells and tissue targeting [90]. Based on nanotechnology, the use of biomaterials as drug carriers to the lungs could help to overcome these limitations [91]. For example, andrographolide has a certain anti-inflammatory effect, but its short biological half-life degradation under acidic and alkaline conditions in the gastrointestinal tract limits its anti-asthmatic potential. The researchers prepared andrographolide-loaded nanoparticles and compared the difference between oral and pulmonary routes of administration. The results showed that only intra-airway administration showed significant efficacy. The as-prepared nanoparticles administrated by pulmonary routescould inhibit IL-4, IL-5, and IL-13 levels in broncho-alveolar lavage fluid and serum IgE content [41]. In another study, Saheli et al. developed the chrysin-loaded nanoparticles (CHR-NPs), and chrysin exhibited a slow and long release at pH 7. In vitro experiments showed a time-dependent accumulation of CHR-NP in A549 cells (Figure 5A). In vivo, lung tissue histology experiments demonstrated that CHR-NP were more effective than free chrysin treatment (Figure 5B). CHR-NP exerted anti-asthmatic effects by inhibiting the activation of the TLR/NF-κB/NLRP3 pathway and thereby reducing the production of pro-inflammatory cytokines in the lung [49]. Additionally, Oliveira et al. determined the extract of pomegranate with PLGA could inhibit eosinophils recruitment to bronchoalveolar fluid. The use of PLGA to deliver pomegranate extract is a promising approach to the treatment of asthma, offering the advantage of reduced single-use doses and sustained release of the drug over time [42].

In addition, PLGA has applications in the treatment of asthma for the delivery of nucleic acids and proteins. Airway-specific defense mechanisms, such as the mucociliary layer, macrophages, and enzymatic activity, make protein administration difficult [92]. Nasal administration of nanosized proteins can avoid first-pass effects on the liver and overcome the limitation of low permeability across mucosal barriers, enabling efficient delivery of proteins into the trachea down to the deep lungs. Unmethylated CpG-oligonucleotides (CpG-ODNs) therapy can effectively induce Th1 immunity and down-regulates established Th2 responses, thereby altering the immune responses to alleviate allergic reactions in asthma [93,94]. Only high doses of CpG-ODN were ingested to produce the therapeutic effects [95]. In response to this shortcoming, the researchers used PLGA-based NPs with CpG-ODN and micro house dust mite (HDM) to make a nanovaccine, and the number of lung eosinophils was significantly reduced after use. This approach provided the opportunity to simultaneously deliver allergen/antigen and CpG-ODN to the same antigen-presenting cells [93,96]. Tumor necrosis factor alpha-induced protein 3 (TNFAIP3/A20) can regulate the functions of various immune cells and is involved in the maintenance of immune homeostasis. The nanovaccine prepared by encapsulating A20/OVA with PLGA, can be stably delivered to specific immune targets and induce the desired immune response in host cells, significantly suppressing the type of Th2 inflammatory response and promoting the production of Treg cells [47].

**Figure 6:**
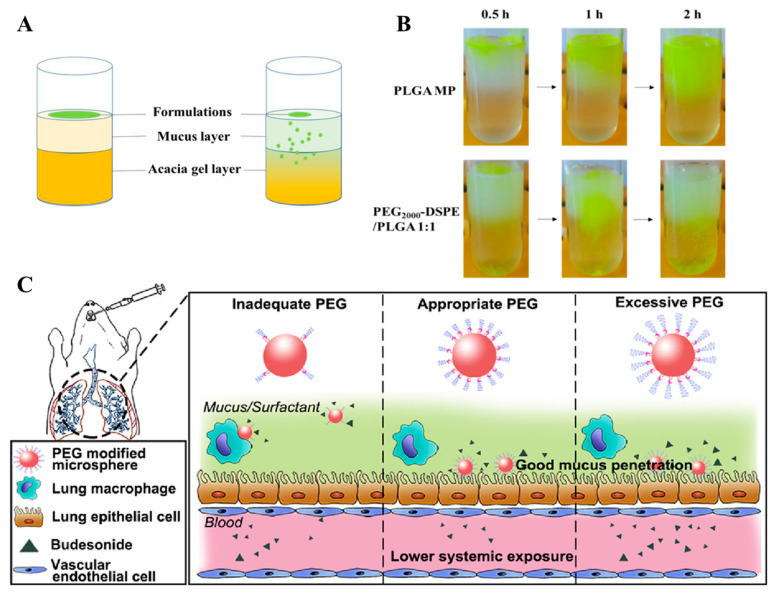
(**A**) Schematic illustration of the mucus penetration study. (**B**) Mucus penetration visualization of the different sample groups. (**C**) Appropriate or excessive PEG could simultaneously overcome the mucus barrier and macrophage uptake. Reproduced with permission from [50] © 2022 Acta Materialia Inc.

PLGA can be modified to change the structure, packaging profile, and drug release kinetics of the prepared nanoparticles to suit the different applications of nanomaterials [86]. Polyethylene glycol (PEG) is the most commonly used hydrophilic modified copolymer because of its good biocompatibility, water dispersibility, stability and easy grafting or adsorption onto the surface of PLGA [86].In addition, the hydrated layer of PEG chains can effectively prevent the recognition and binding of tonin proteins to plasma proteins, and reduce the phagocytosis of reticuloendothelial system (RES), thus increasing the stability of drug-loaded nanoparticles and enhancing the blood circulation half-life [97,98]. Low molecular weight heparin (LMWH) particles prepared with PEG-PLGA dimer (from 47.37 ± 6.02 μm to 21.35 ± 3.60 μm) could effectively improve the airway wall thickness, mainly due to the relatively low density and good aerodynamic behavior of the large porous particles, which improved the deposition efficiency in the lung [43]. In addition, PLGA-PEG-based microspheres are one of the promising approaches to overcome barriers to mucus and alveolar macrophage uptake deep in the lungs. Li et al. prepared budesonide-loaded porous PLGA microparticles and found that the microspheres prepared with PEG_2000_ had a strong ability to cross the mucus layer (Figure 6A,B) [50]. Furthermore PLGA microspheres prepared with appropriate or excessive PEG_2000_ could evade macrophage uptake (Figure 6C). Another study showed that the PEG_5000_ layer could promote the rapid penetration of bavachinin-loaded PLGA NPs through the mucosal surface of the gastrointestinal tract, prolonging the circulation time of low water-soluble bavachinin in the blood [45,99].

In addition, chitosan can also modify PLGA nanoparticles. After CS modification, the biocompatibility of the nanoparticles and the adhesion to protein molecules were enhanced, promoting their retention in the lung. For example, the Ca^2+^/calmodulin-dependent protein kinase (CaMKII) can regulate the reactive oxygen species (ROS) production released by neutrophils in the asthma airways. To improve the in vivo bioavailability of CaMKII, the researchers prepared CaMKIIN-loaded nanoparticles using CS and PLGA for the delivery of CaMKII [39]. Oropharyngeal installation of CaMKIIN-loaded NPs could reduce the mucus production and airway hyperresponsiveness in asthmatic mice. Moreover, the uptake by lung cells were increased after CS modification.

**Figure 7:**
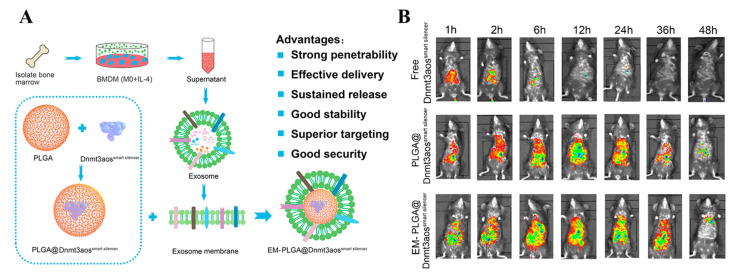
(**A**) The schematic representation of EM-PLGA@Dnmt3aos^smart silencer^. (**B**) In vivo tracking and tissue distribution of EM-PLGA@Dnmt3aos^smart silencer^ in mice. Reproduced with permission from [51] © 2022 Elsevier B.V.

Phospholipids have also been used to modify the surface of PLGA nanoparticles due to the presence of electrostatic interactions. The recently developed lipid vesicles derived from native cell membranes endowed the surface of PLGA nanoparticles with unique cell mimicking characteristics. Pei et al. constructed PLGA nanoparticles for loading DNA methyltransferase 3A (Dnmt3aos); a smart silencer (EM-PLGA@Dnmt3aos^smart silencer^) was constructed after exosome membrane (EM) modification (Figure. 7A) [51]. Dnmt3aos is the long non-coding RNAs (lncRNAs), which are differentially expressed in M1/M2 polarized bone marrow-derived macrophages. EM-PLGA@Dnmt3aossmart silencer was shown to target lung macrophages and exerted therapeutic effects by regulating the polarization of M2 macrophages. In vivo experiments showed excellent biocompatibility and targeting capability (Figure 7B). The surface modification of lipid vesicles endows the nanoparticles with biomimetic and biodegradable properties, increasing the possibility of further surface modifications and improving the targeting of the drug, thus enhancing the therapeutic efficacy.

#### 2.1.3. Application of PAMAM in Asthma

Dendrimers consist of a group of macromolecules with carefully tailored structures, controlled by an iterative synthesis and considered as nano-size units with tree-like branches [100,101]. Three things determine their properties: the polymeric branches that emerge from the core, the repeating units that determine the solubilization ability, and the end groups that determine the behavior of the dendrimer [53]. There are several types of dendrimers, such as PEI, carbosilane, and PAMAM dendrimers [101]. However, PAMAM dendrimers have NH_2_ terminal functions, thus can be easily modified according to the designed structure, and are considered to be the most flexible dendrimers for applications, especially in drug delivery and biological applications [100,102]. In recent years, nanocarrier systems for pulmonary delivery of PAMAM dendrimers have been increasingly reported regarding their safety for use with respiratory tissues and enhanced drug absorption by lung cells, indicating the potential use of dendrimers in pulmonary delivery [103,104].

PAMAMs dendrimers for asthma therapy usually require modification of cationic groups on their surface to neutral or anionic moieties to avoid toxicity and liver accumulation [105]. Cytotoxicity could be reduced by lauroyl chains and polyethylene glycol, which usually helped shield excess positive charge [102,106]. Lauroyl chains and polyethylene glycol are used with PAMAM dendrimers to prepare nanoparticles for lung delivery, which could enhance the safety of respiratory tissue and the absorption of drugs by lung cells, and improve the safety of dendrimer delivery in the lung [107]. Modified PAMAM dendrimers can be used as carriers for the delivery of various biomolecules such as nucleic acids and proteins [108,109]. The surface groups of amine-terminated PAMAM dendrimers are protonated under physiological conditions [110]. The surface groups of amine-terminated PAMAM dendrimers can bind to nucleic acids, forming nanoscale complexes, which are able to offer the chance to improve targeting of siRNA to the airway epithelium cell cytosol [52]. PAMAM dendrimers have high aqueous solubility and easily modified surface functional groups, which are commonly used for the solubilization of hydrophobic drugs for asthma, such as dexamethasone, rifampicin methylprednisolone, and beclomethasone dipropionate [53,54,111]. After binding to PAMAM dendrimers, these drugs significantly enhanced the drug accumulation in the lungs and their bioavailability.

#### 2.1.4. Application of PHPMA in Asthma

PHPMA is a structurally stable, non-immunogenic, and highly hydrophilic polymer that can be metabolized in the human body, and has been extensively used over the last few decades [112]. PHPMA drug conjugates have many advantages compared with free drugs: longer retention time in the body, safer systemic administration, better therapeutic efficacy and better metabolization [113,114]. Most of these PHPMA-antibody conjugates are prepared by aminolysis of the terminal functional amino group of the polymer to modify the antibodies at the end of the polymer chain, or by polymerization of the N-(2-Hydroxypropyl) methacrylamide (HPMA) copolymer with an antibody-containing monomer. In these systems, the PHPMA backbone is modified with biodegradable oligopeptide side chains, and the ends are modified with targeting antibodies and drugs, and are randomly distributed along the backbone [115]. Such adhesins can be designed as protein delivery systems or drug delivery systems for easier targeted drug delivery. Polymeric drug conjugates based on water-soluble copolymers of HPMA have been extensively studied as a class of drug delivery systems [116]. For example, Moog et al. developed a novel polymeric cell adhesion inhibitor, a PHPMA-embedded sialyl-LewisX (SLeX)-polymer system. SLeX, a natural ligand for selectins, cannot be an anti-inflammatory agent because it is subject to rapid digestion by glycosidases and peptidases in the blood. However, the SLeX-polymer system reduced methacholine-induced AHR in mice and reduced macrophage migration to the endothelium, which had therapeutic potential for allergic airway inflammation [55].

### 2.2. Application of Lipid-Based Carriers in Asthma

Solid lipid nanoparticles are a new generation of nanoparticle drug delivery systems made by adsorbing or encapsulating drugs in lipid membranes [117]. Solid lipid nanoparticles are made up of biodegradable and safe lipid components such as solid natural or synthetic lipids such as lecithin, triacylglycerols etc. The distinguishing feature of solid lipid nanoparticles is their ability to carry a wide range of therapeutic agents, including small drug molecules, large biomolecules, genetic material and vaccine antigens [118]. Solid lipid nanoparticles are emerging as drug delivery systems with good physical stability, protection against environmentally sensitive unstable drugs and targeted drug delivery, which can be used for targeted drug delivery.

Rhynchophylline is the main alkaloid in the plant medicine *Uncaria*, a monomer with anti-inflammatory effects [119]. The formulate rhynchophylline-solid lipid nanoparticles (Rhy-SLNs) showed that water solubility of phynchophylline was significantly improved and the encapsulated phynchophylline massively aggregated in the lung. The encapsulated phynchophylline could reduce airway inflammation and oxidative stress by inhibiting the p38 signaling pathway [120]. Not surprisingly, the encapsulated proanthocyanidins also showed better therapeutic effects than free proanthocyanidins [121]. Additional studies have shown that solid lipid nanoparticles could deliver curcumin. In an ovalbumin (OVA)-induced mouse model of asthma, airway constriction was significantly inhibited, and airway inflammation was reduced on the basis of accumulation in the lungs. The study further showed that curcumin-solid lipid nanoparticles (curcumin-SLNs) reduced the expression of IL-4 and IL-13 in the lungs of asthmatic mice by inhibiting the activation of NF-κB and alleviated the occurrence of asthma [121,122]. Solid liposome nano-delivery systems have the advantages of good cytocompatibility, proven in vivo efficacy, good lipid bilayer penetration, and safety. Solid lipid NPs are also highly physically stable and can be stored at 2–8 °C for up to 3 months, allowing for a wide range of applications [123].

### 2.3. Application of Extracellular Vesicle-Associated Carriers in Asthma

In recent years, extracellular vesicles, natural nanomaterials secreted by cells, have been developed for asthma treatment as they mediate intercellular communication in response to the immune system [124]. Extracellular vesicles (microvesicles and nanoscale exosomes) can deliver some proteins and nucleic acids for asthma treatment, and some extracellular vesicles themselves can distinguish different types of allergies in patients and have the potential to treat asthma [125,126,127]. Exosomes with low immunogenicity and high safety have the potential to penetrate the pores of the mucus layer and deliver therapeutic agents directly to airway epithelial cells [128]. With the astonishing increase in the number of studies on extracellular vesicles in recent years, it is clear that there is an exciting field in asthma treatment. Biocompatible extracellular vesicles can deliver labile genes or proteins to inflamed lungs. These studies will make a huge contribution to asthma treatment.

Adipose-derived mesenchymal stem cells (ADSCs) with low immunogenicity can promote cell communication by secreting exosomes [129]. MiR-301a-3p, a newly reported miRNA in ADSC exosomes, plays an anti-inflammatory role by regulating PI3K signaling and causing the polarization of macrophages from M1 pro-inflammatory cells to M2 anti-inflammatory cells [130]. The researchers found that MiR-301a-3p could inhibit the proliferation and migration of airway smooth muscle cells caused by platelet-derived growth factors by inhibiting the signal transducer and activator of the transcription 3 (STAT3) pathway, so as to achieve the purpose of asthma treatment [131]. Studies have shown that targeted inhibition of STAT3 activity was closely related to IL-17 and airway inflammation and could be used as a new target for treating severe asthma [132]. Furthermore, mmu-circ-0001359 produced by ADSCs-derived exosomes reduced airway remodeling by enhancing FoxO1 signaling and activating M2-like anti-inflammatory macrophages [133]. In addition to ADSCs, extracellular vesicles secreted by dendritic cells as antigen-presenting cells also could regulate adaptive immunity [134,135]. Wu et al. showed that heme-induced dendritic cell generation of extracellular vesicles could reduce the number of inflammatory cells in the lung, and also inhibited the secretion of inflammatory cytokines such as IL-4 and IL-13 to achieve the purpose of asthma treatment [136].

Although extracellular vesicles have the advantages of good cytocompatibility and non-toxicity, their small yield and high price also hinder their development. Oxygen concentration plays an important role in the proliferation of mesenchymal stem cells and the secretion of extracellular vesicles [137]. Hypoxic treatment of mesenchymal stem cells can improve extracellular vesicle secretion and is promising in various disease models [138]. Dong et al. placed the mesenchymal stem cells in a state of hypoxia, and found that this condition allowed the cells to secrete more high-quality extracellular vesicles [127]. The hypoxic treatment strategy not only provided exosomes for asthma treatment but also proposed a constructive solution to the low production of exosomes [127]. In addition to improving the yield of extracellular vesicles, another study demonstrated the development of standard homogeneous extracellular vesicles and the purification of extracellular vesicles using an anion exchange chromatography protocol. Purified mesenchymal stem cell extracellular vesicles suppressed innate lymphoid cells (ILC2) levels and reduced lung inflammatory cell infiltration and mucus production [128].

In recent years, more and more studies have been conducted on extracellular vesicles as nanoparticle carriers. Extracellular vesicles hold promise as therapeutics for diseases and as excellent delivery platforms. Exosomes as a type of extracellular vesicles, can deliver exogenous siRNA or miRNA to target disease sites in vivo, regulate gene expression, and play the therapeutic roles [139,140]. Due to the distinct differences in miRNA expression profiles between normal and asthmatic lung tissues, the delivery of miRNA inhibitors is considered a novel therapy for asthma [141,142]. Epithelial-mesenchymal transition, which occurs in airway remodeling, is closely related to asthma. miR-21-5p is considered a biomarker of airway reversibility [143]. Li et al. used fluorescently labeled exosomes to achieve airway epithelial cell-targeted delivery of miR-21-5p inhibitors [144]. The results proved that miR-21-5p-containing exosomes could promote epithelial-mesenchymal transition by targeting the transforming growth factor beta1 (TGF-β1) pathway of recombinant mothers against decapentaplegic homolog 7 (Smad7) to achieve the therapeutic effect [145].

### 2.4. Application of Metal Nanoparticles in Asthma

Metal nanoparticles have not only been used as catalysts in the field of chemistry but also have made breakthroughs in the field of medicine. Metal nanoparticles have been explored in the biomedical field due to their advantages, such as reducing excess ROS in the airways of asthma [146]. In recent years, silver nanoparticles have been explored in the treatment of asthma. Silver nanoparticles inhibited the expression of the oversecreted mucin 5AC (Muc5ac) during asthma development and significantly inhibited phosphatidylinositol 3-kinase (PI3K) and phosphorylated Ak strain transforming (Akt) levels in the airways. Its mechanism of action might be to achieve an anti-inflammatory effect by inhibiting the PI3K signaling pathway [147]. In addition, another study demonstrated that the anti-inflammatory effect of silver nanoparticles might be through the NF-κB signaling pathway. Silver nanoparticles could also significantly inhibit the highly expressed ROS in the lungs of asthmatic mice and attenuate antigen-induced airway inflammation and hyperresponsiveness [148]. The outstanding antioxidant capacity of silver nanoparticles hold great promise in treating asthma. In addition, silver nanoparticles made outstanding contributions to the formation of airway scaffolds. The airway scaffolds constructed by silver nanoparticles with antiproliferative activity and cisplatin improved the surface function of traditional scaffolds [149]. Su et al. conducted a proteomic study to explore the differences between healthy and asthmatic individuals who inhaled silver nanoparticles and found that silver nanoparticles could regulate multiple pathways, including coagulation, chemokine-mediated inflammation, and T lymphocytes (T-cell) activation. Bronchoalveolar lavage (BALF) proteomic analysis of differential proteins in healthy and asthmatic individuals could be used as an efficient method for screening silver nanoparticle adjuvants [150].

In addition, compared with silver nanoparticles, gold nanoparticles have lower toxicity, and have gained attention in recent years [151,152,153]. Kang. observed the morphology and microstructure of gold nanoparticles. When OVA-induced asthma mice were treated with gold nanoparticles, the macrophages loaded with gold nanoparticles migrated to the target lung tissue and exerted anti-inflammatory effects by inhibiting inflammatory mediators such as inducible NO synthase (iNOS), cyclooxygenase-2 (COX-2), and nitric oxide (NO) [154]. Another study demonstrated that gold nanoparticles could modulate lung function in mice, improve leukocyte infiltration, and reduce mucus secretion levels and cytokine production. The mechanism of action might be related to the down-regulation of oxidative stress levels in the lung [155]. Although the anti-inflammatory effect of gold nanoparticles may be beneficial for asthma treatment, it is also important to consider whether systemic absorption may cause side effects. In order to improve the mucus penetration and intrapulmonary targeting of gold nanoparticles, Omlor et al. prepared polyethylene glycol gold nanoparticles, avoiding the adverse reactions caused by systemic absorption [156].

In addition to the nanoparticles mentioned above, ultra-small-sized superparamagnetic iron oxide, (SPIO) nanoparticles have received great interest for their low intrinsic toxicity, easy coupling to target groups, surface functionalization and ease of detection. Wu et al. demonstrated that DiR-SPION conjugated to the anti-suppression of tumorigenicity 2 (ST2) blocking antibodies (anti-ST2 NPs) significantly alleviated airway inflammation by reducing the IL-33 and IL-13 levels and the percentage of CD4+T cells, and efficiently suppressed the development of asthma by blocking the function of group 2 innate lymphoid cells (ILC2s) [157]. Furthermore, hollow mesoporous silica nanoparticles (HMSNs) with good biocompatibility and low cytotoxicity are promising inorganic nanocarriers due to their large surface area, adjustable pore size and well-defined surface properties. Xia et al. loaded the major house dust mite (HDM) allergen (Der f2) onto HMSNs, which act as vaccine carriers, to achieve a high degree of allergic asthma prevention with reduced local side effects [158,159]. Moreover, metal organic frameworks (MOFs) have a large specific surface area, clear structure, adjustable pore structure, high porosity and regular shape, etc. As a new type of nanomaterial, it has a late start in the biomedical field but is developing rapidly and is a promising nanomaterial for future asthma treatment [160]. Besides, cerium oxide nanoparticles, a type of nanoparticle capable of performing enzymatic functions, called “nanoenzymes”, which inhibit the over-activation of signal pathways such as NF-κB, could be an ideal anti-inflammatory carrier in future asthma treatments [161].

## 3. Discussion and Prospects

Currently, inhaled glucocorticoids are the mainstay treatment strategy for asthma treatment, however, long-term use can cause drug resistance and many side effects, such as osteoporosis, growth suppression in children, and diabetes. In recent years, new drugs have been developed to combat asthma, and the delivery efficiency of existing inhaled drugs has been improved to increase the bioavailability and effectiveness of delivered drugs. As an interdisciplinary science, nanotechnology has been widely used in the research of asthma treatment and has produced good therapeutic effects. Although well-appreciated results have been achieved, there are still many issues to be resolved.

At first, the preparation process of some nanoparticles is complex, high-priced, and has poor repeatability, making some nanoparticles difficult for large-scale production for clinical applications [162,163]. Furthermore, properties such as composition, size, shape and zeta potential of the nanoparticles have significant impacts on pulmonary drug delivery [91]. These determined whether the nanoparticles could pass through the thickened mucus layer of asthmatic patients. In addition, further attention needs to be paid to drug release of that encapsulated in nanoparticles, such as the burst release and leakage from nanoparticles, which can affect the stability of nanoparticles [164]. Last, but not least, the accumulation of nanoparticles in the lungs is a cause for concern. In the design of nanoparticles, the use of non-metabolizable nanoparticles in the lung should be avoided. As a participant in asthma treatment, the interaction between nanomaterials and the endotracheal microenvironment should also be considered to deal with asthma more effectively.

There are some solutions to the above problems. First, it is necessary to use low toxic carriers to prepare nanoparticles and simplify the preparation process to achieve large-scale production [127]. In addition, nanoparticles with positive charges properties are not good at penetrating airway mucus effectively [165,166], and the cationic carriers are often required to be modified on their surfaces to have neutral or anionic groups in order to avoid toxicity. In order to control the release of the drugs, nanoparticles could be designed to have targeted response capabilities, such as sensitivity and inflammation sensitivity, and drugs can be released under specific conditions. For example, nanoparticles modified with pH-sensitive hydrophobic segments were prepared in pH-responsive nanoparticles for targeted release in inflamed lungs [167,168]. Finally, in order to allow the nanoparticles to penetrate the mucus layer and to better improve the retention time in the lungs, nanoparticles can be designed be smaller, to have targeted molecules, to be electrically neutral, to have high encapsulation efficiency, and to have low toxicity [169]. Materials with mucoadhesive properties, such as CS, can be used in the nanoparticles to enhance the retention of the nanoparticles in the lungs. Importantly, the surface of the nanoparticles can be modified with PEG. Studies have shown that PEG-modified nanoparticles can easily pass through the mucus layer [170,171].

Most advances in asthma treatment in recent years have focused on new therapies such as precision medicine, endotypes and phenotypes, biomarkers and biologics. Pre-cision medicine is well suited to the heterogeneity and complex pathogenesis of asthma, which requires a good understanding of asthma biomarkers, phenotypes, endotypes, genotypes and regional patterns. Personalized therapeutic approaches and targeted therapy for asthma patients promise to be the future of asthma treatment. Nanotechnology promises to be a great addition to targeted asthma therapy by controlling the targeted release of drugs. In future studies, researchers may be able to provide patients with personalized and precisely individualized treatment targeting drug release by understanding the immunology of asthma. For example, nanoparticles-based drugs targeting eosinophil release may be benefit for patients with Th2-high subtype asthma, or those targeting neutrophils release for Th2-low subtype asthma patients, which can enhance the effectiveness of treatment asthma.

Although many preclinical studies have focused on nanotechnology as a potential alternative for treating asthma, no clinical studies have been conducted. However, there have been the clinical trials for lung damage caused by COVID-19 (NCT04397510) [91]. Therefore, nanotechnology is expected to be used to treat asthma from the laboratory to the clinic. In this process, there are still many difficulties to overcome. It is essential to develop nanomaterials suitable for pulmonary drug delivery for the treatment of asthma and to further study the anti-asthma mechanism of nanomaterials. In conclusion, this paper reviewed new strategies for the treatment of asthma, with a focus on the use of polymeric nanoparticles in asthma treatment, and discussed some of the issues and future research directions. Although some progress has been made, more economical and simpler experimental methods still need to explore more economical and simpler experimental methods to alleviate the dilemma of asthma treatment and to enter clinical trials as early as possible.

## Figures and Tables

**Table 1:** Summary of the application of polymers in asthma.

Nanocarriers	Functional Component	Size	Cell Lines/Animal Models	Ref.
CS	Baicalein	285 ± 25 nm	BALB/c mice	[31]
CS	BCG-polysaccharide nucleic acid and ovalbumin	1130 ± 22 nm	BALB/c mice	[32]
CS	Heparin	359 ± 21 nm	Rat Mast Cells	[33]
CS	Budesonide	551 ± 7 nm	BALB/c mice; Rats	[34]
CS	Interleukin-17 receptor C	212.2 nm	BALB/c mice	[35]
CS	Hyaluronic acid	164.2 ± 9.7 nm	BALB/c mice	[36]
CS	Levosalbutamol sulphate	/	/	[37]
CS	Tris(2-carboxyethyl) phosphine (TCEP)	128.0 ± 2.1 nm	NIH 3T3 cells; A549 cells; L929 cells; BALB/c mice	[38]
PLGA/CS	CaMKII inhibitor peptide	230 nm	HAECs cells; MTBEC cells; BALB/c mice	[39]
PLGA	Budesonide	8.2 ± 1.5 μm	BALB/c mice	[40]
PLGA	Andrographolide	205 nm	C57 BL/6 mice	[41]
PLGA	pomegranate encapsulated extract	7.22 μm	BALB/c mice	[42]
PEG-PLGA	Low-Molecular-Weight Heparin	47.37 ± 6.02 μm	BALB/c mice	[43]
PLGA	Montelukast	1.59–2.51 μm	Calu-3 cells; BALB/c mice	[44]
PEG-PLGA	Bavachinin	196 nm	HeLa cells; NIH-3T3 cells; BALB/c mice	[45]
PLGA	Salbutamol	8.24 µm	A549 cells	[46]
PLGA	A20-OVA	100–250 nm	/	[47]
PLGA	Curcumin	2.5 ± 0.4 µm	BALB/c mice	[48]
PLGA	Chrysin	99.034 ± 9.494 nm	A549 Cells	[49]
PEG-PLGA	Budesonide	3.46 ± 0.05 µm	Rats	[50]
PLGA	Dnmt3aos smart silencer	137 ± 4.5 nm	M2 macrophages; C57BL/6 mice	[51]
PAMAM	G4NH2-siRNA complexes	254 ± 52 nm	A549 cells	[52]
PAMAM	Beclometasone dipropionate	1.68–5.82 µm	/	[53]
PAMAM	Dexamethasone	/	BALB/c mice	[54]
PHPMA	P-selectin antagonist	30–400 nm	BALB/c mice; C57BL/6 mice	[55]
